# *Cryptosporidium parvum* hijacks a host’s long noncoding RNA U90926 to evade intestinal epithelial cell-autonomous antiparasitic defense

**DOI:** 10.3389/fimmu.2023.1205468

**Published:** 2023-06-05

**Authors:** Marion L. Graham, Min Li, Ai-Yu Gong, Silu Deng, Kehua Jin, Shuhong Wang, Xian-Ming Chen

**Affiliations:** ^1^ Department of Microbial Pathogens and Immunity, Rush University Medical Center, Chicago, IL, United States; ^2^ Department of Medical Microbiology and Immunology, Creighton University School of Medicine, Omaha, NE, United States; ^3^ Department of Biochemistry and Molecular Biology, School of Basic Medicine, Hubei University of Science and Technology, Xianning, Hubei, China

**Keywords:** lncRNA, *Cryptosporidium*, intestinal epithelium, defense, Aebp1, CSpV1, U90926

## Abstract

*Cryptosporidium* is a zoonotic apicomplexan parasite that infects the gastrointestinal epithelium and other mucosal surfaces in humans. It is an important opportunistic pathogen in AIDS patients and a leading cause of infectious diarrhea and diarrheal-related death in children worldwide. The intestinal epithelial cells provide the first line of defense against *Cryptosporidium* infection and play a central role in activating and regulating the host’s antiparasitic response. Increasing evidence suggests that long noncoding RNAs (lncRNAs) participate in host-pathogen interactions and play a regulatory role in the pathogenesis of diseases but the underlying molecular mechanisms are not fully understood. We previously identified a panel of host lncRNAs that are upregulated in murine intestinal epithelial cells following *Cryptosporidium* infection, including U90926. We demonstrate here that U90926 is acting in a pro-parasitic manner in regulating intestinal epithelial cell-autonomous antiparasitic defense. Inhibition of U90926 resulted in a decreased infection burden of the parasite while overexpression of U90926 showed an increase in infection burden in cultured murine intestinal epithelial cells. Induction of U90926 suppressed transcription of epithelial defense genes involved in controlling *Cryptosporidium* infection through epigenetic mechanisms. Specifically, transcription of Aebp1, which encodes the Aebp1 protein, a potent modulator of inflammation and NF-κB signaling, was suppressed by U90926. Gain- or loss-of-function of Aebp1 in the host’s epithelial cells caused reciprocal alterations in the infection burden of the parasite. Interestingly, *Cryptosporidium* carries the *Cryptosporidium* virus 1 (CSpV1), a double-stranded (ds) RNA virus coding two dsRNA fragments, CSpV1-dsRdRp and CSpV1-dsCA. Both CSpV1-dsRdRp and CSpV1-dsCA can be delivered into infected cells as previously reported. We found that cells transfected with *in vitro* transcribed CSpV1-dsCA or CSpV1-dsRdRp displayed an increased level of U90926, suggesting that CSpV1 is involved in the upregulation of U90926 during *Cryptosporidium* infection. Our study highlights a new strategy by *Cryptosporidium* to hijack a host lncRNA to suppress epithelial cell-autonomous antiparasitic defense and allow for a robust infection.

## Introduction

*Cryptosporidium* is a protozoan parasite that infects the gastrointestinal epithelium and other mucosal surfaces in humans and animals (1). It is an important opportunistic pathogen in AIDS patients and is the second most common pathogen responsible for moderate to severe diarrhea in children under 2 years of age in developing countries (2). Infection with *Cryptosporidium* shows a significant association with mortality in children and appears to predispose them to lasting defects in body growth and cognitive ability (2–5). The majority of human *Cryptosporidium* infections are caused by two species: *C. parvum* and *C. hominis* (6, 7). Waterborne transmission grants the parasite the potential to cause large-scale outbreaks and epidemics in both developing and developed nations (8–10). After ingestion of *Cryptosporidium* oocysts from contaminated water or foods, the oocysts are excysted in the gastrointestinal tract to release infective sporozoites. The sporozoites then attach to the apical membrane of the intestinal epithelial cells and form an intracellular but extracytoplasmic vacuole where the organism remains and further develops (6, 11). Therefore, epithelial cells play a central role in activating and orchestrating both innate and adaptive anti-*Cryptosporidium* responses (12, 13).

Epithelial cells along the mucosal surface provide the first line of defense against luminal pathogen infections such as *Cryptosporidium*. These epithelial cells are an important component of digestive mucosal immunity and represent an integral component of a highly regulated communication network involving essential signals to and from the underlying cells of the gastrointestinal mucosa (14). Upon microbial challenge, gastrointestinal epithelial cells initiate a series of immune reactions including the release of inflammatory cytokines/chemokines and the production of antimicrobial molecules. These molecules then activate immune effector cells and mobilize them to the site of infection (15). Among these signaling pathways activated by *C. parvum* infection is the TLR4/nuclear factor kappa B (NF-κB) signaling pathway which results in the production and secretion of those cytokines/chemokines which may kill or inhibit parasite growth (16, 17). Nevertheless, the success of the parasite is the result of its ability to evade these host immune responses while still utilizing the host’s resources. The host and parasite partake in a co-evolutionary “arms race” in which the parasite evolves novel strategies to escape the immune response and the immune system simultaneously evolves strategies to kill the parasite. This dynamic competition results in the parasite employing innovative ways of utilizing host resources and machinery (18).

Increasing evidence suggests that a certain portion of the eukaryotic genome is transcribed as non-protein coding RNAs (ncRNAs) including long ncRNAs (lncRNAs) which are noncoding transcripts longer than 200 nt. Thousands of lncRNAs have been identified and the majority of them may be functional (19, 20). LncRNAs function to regulate gene transcription through specific interactions with cellular factors, including proteins, DNA, and other RNA molecules via nucleotide base pairing or the formation of structural domains generated by RNA folding (21–23). Studies have found many of these lncRNAs to be targets of inflammatory pathways and thus their expression is altered during inflammation or microbial infection (24, 25). Several lncRNAs have been shown to be induced in innate immune cells and may play a role in regulating the immune response (26–29). Furthermore, recent evidence suggests that microorganisms can utilize lncRNAs to regulate or evade the host’s immune response. During *chlamydia trachomatis* infection, the host lncRNA MIAT is upregulated and involved in not only resisting apoptosis but also the growth and development of *chlamydia trachomatis* (30). *Toxoplasma gondii* infection results in altered host lncRNA expression to target UNC93B1 expression, impairing the secretion of cytokines IL-12, TNF-α, IL-1β, and IFN-γ, ultimately inhibiting the host immune response (31).

Using an *in vitro* infection model of intestinal epithelial cells by *C. parvum*, a panel of host cell lncRNAs that are upregulated following *C. parvum* infection has been recently identified (25). Among the lncRNAs upregulated was U90926, previously also known as NR_033483, with its gene located on chromosome 5 near XR_880430.2 (32). This lncRNA can be induced in activated macrophages, is protective in endotoxic shock, and may encode a secreted peptide in macrophages (32). In this study, we demonstrate that U90926 displays an inhibitory effect on the innate immune response against *C. parvum* infection by transcriptionally repressing defense gene expression. Interestingly, the upregulation of U90926 itself seemed to be induced by a small double-stranded RNA virus found in *Cryptosporidium* called *Cryptosporidium parvum* virus (CSpV1) (33). Therefore, our data suggest that upon *C. parvum* infection, CSpV1 induces the upregulation of host lncRNA, U90926, which targets transcription of host defense genes and inhibits epithelial antiparasitic response. This study highlights a novel evasion strategy in which *C. parvum* hijacks a host’s lncRNA to suppress epithelial cell-autonomous antiparasitic defense to allow for a robust infection.

## Materials and methods

### *C. parvum* and cell lines

*C. parvum* oocysts of the Iowa strain were purchased from a commercial source (Bunch Grass Farm, Deary, ID). The mouse intestinal epithelial cell line (IEC4.1) was received as a kind gift from Dr. Pingchang Yang (McMaster University, Hamilton, Canada). Culture media were supplied with 10% FBS (Ambion) and antibiotics. Stable IEC4.1 cells with deficient in U90926 were generated through transfection of cells with the CRISPR/Cas9 KO(h) (U90926-CRISPR/Cas9 KO) and the HDR plasmid (U90926-HDR) as previously described (34).

### Infection models and infection assays

Models of intestinal cryptosporidiosis using intestinal epithelial cell lines and 2D monolayers derived from 3D enteroids were employed as previously described (25, 35). Infection was done in culture medium (DMEM-F-12 with 100U/ml penicillin and 100µg/ml streptomycin) containing viable *C. parvum* oocysts after treatment with 1% sodium hypochlorite. Cells were then cultured for 4 hr at 37°C for attachment and invasion by the parasites. After extensive washing with DMEM-F-12 medium three times to remove free parasites. Cells were cultured for additional time periods. Intestinal epithelium and 3D enteroids were employed as previously described (35). Briefly, the small intestine was opened longitudinally and washed with ice-cold Ca2+ and Mg2+ free PBS before being cut into 1-2 mm fragments and washed with ice-cold Ca2+ and Mg2+ free PBS 3 times. The cut fragments were incubated in ice-cold 2 mM PBS/EDTA at 4° C for 30 min with gentle rotation followed by vigorous shake until the PBS solution was mostly opaque with dislodged crypts and villus particles. Large tissue fragments were removed through a 100-µm cell strainer (Becton-Dickinson Bioscience, Franklin Lakes, NJ). The pass-through was centrifuged 150 g for 5 min at 4°C and the pellet was collected as the intestinal epithelium. 2D monolayers derived from 3D enteroids were cultured as previously described (35–37) and exposed to *C. parvum* infection for 24-48 hr.

For *in vivo* experiments, a well-developed infection model of cryptosporidiosis in neonatal mice was used (38, 39). Briefly, 5 days after birth, mice received *C. parvum* oocysts by oral gavage (10^6^ oocysts per mouse) to develop intestinal cryptosporidiosis. Mice that received PBS by oral gavage were used as controls. At 48- and 72 hr after *C. parvum* or PBS administration, animals were sacrificed, and ileum epithelium tissues were obtained for biochemical analysis and infection assessment as previously reported (25, 35, 40, 41). All research studies involving the use of animals were reviewed and approved by the Institutional Animal Care and Use Committees of the Rush University Medical Center and were carried out in strict accordance with the recommendations in the Guide for the Care and Use of Laboratory Animals.

### Quantitative real-time PCR

For quantitative analysis of RNA and *C. parvum* RNA expression, qRT-PCR was performed as previously reported (35, 40–43), using the SYBR green PCR master mix (Applied Biosystems, Carlsbad, CA, USA). Briefly, total RNA was isolated and possible remaining DNA was removed using TRI reagent and treated with a DNase-free kit (Ambion, MA, USA). qRT-PCR was then performed using 25 ng of template cDNA from reverse transcription for each RNA gene of interest. The expression level of each RNA was calculated using the threshold cycles (ΔΔCT) method and normalized to glyceraldehyde-3-phosphate dehydrogenase (*Gapdh*). All sequences of PCR primers are listed in **Table S1**.

### Transfections with siRNAs

Custom-designed RNA oligonucleotides against U90926 and a scrambled RNA (used as the control) were synthesized by IDT (Integrated DNA Technologies, Coralville, IA) and transfected into cells with the Lipofectamine RNAiMAX according to the manufacturer’s protocol (Invitrogen). Sequences of siRNAs were GAGGTTCTGTGAATTCTTTAA for U90926 and UUCUCCGAACGUGUCACGUUU for the control.

### Chromatin immunoprecipitation and chromatin isolation by RNA purification

Chromatin immunoprecipitation (ChIP) assays were performed as previously described (25, 28, 29) using the ChIP Assay Kit (Millipore Sigma) in accordance with the manufacturer’s protocol. An anti-H3K4m1 (Abcam) was used and the specific primers are listed in **Table S1**. Chromatin isolation by RNA purification (ChIRP) analysis was performed as previously reported (44). Briefly, glutaraldehyde cross-linked for chromatin isolation and a pool of tiling oligonucleotide probes with an affinity specific to the U90926 sequence were used. The sequences for each probe are listed in **Table S1**. The DNA sequences of the chromatin immunoprecipitates were confirmed by qRT-PCR using the same primer sets covering the gene promoter regions of interest as for ChIP analysis. A pool of scrambled oligonucleotide probes for LacZ (**Table S1**) was used as a control.

### Luciferase reporter constructs and luciferase assay

The 266-1000 region of the *U90926* gene, which contains five putative NF-kB binding sites, was cloned into the multiple cloning sites of the PNL1.1 NLUC vector (Fisher Scientific). The primers to amply the sequence are listed in **Table S1**. An empty vector was used as a control. Cultured IEC4.1 cells were transfected with the reporter construct overnight with or without *C. parvum* infection followed by an assessment of luciferase activity. The luciferase activity was normalized to the control β-galactosidase level and compared to the empty vector.

### Statistical analysis

All values are given as mean ± SEM. The means of the groups were from at least three independent experiments and compared with Student’s t-test (unpaired) or the ANOVA test when appropriate. P values <0.05 were considered significant.

## Results

### Upregulation of U90926 expression in intestinal epithelium following *C. parvum* infection

**Figure 1:**
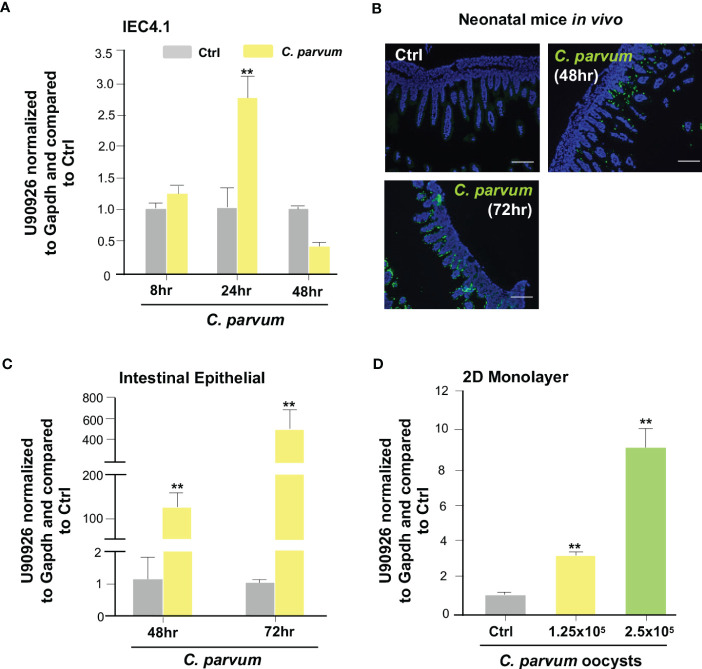
Upregulation of U90926 expression in intestinal epithelium following *Cryptosporidium parvum* infection. **(A)** Upregulation of U90926 in cultured intestinal epithelial cells (IEC4.1) following *C. parvum* infection. IEC4.1 cells were exposed to *C. parvum* infection for 8-48 hr and expression of U90926 was measured via qRT-PCR. **(B)** *C. parvum* infection of intestinal epithelium in neonatal mice. Indirect immunofluorescent staining of the ileum confirms *C. parvum* infection. **(C)** Upregulation of U90926 in murine intestinal epithelium following *C. parvum* infection *in vivo*. Neonates of mice (5 days old) were infected via oral administration for 48-72 hr. qRT-PCR was used to measure expression levels of U90926. **(D)** Upregulation of U90926 in 2D intestinal epithelial monolayers following *C. parvum* infection. 2D monolayers were exposed to *C. parvum* infection (at two different parasite oocyst doses: 1.25 X 10^5^ and 2.5 X 10^5^ oocysts/well) for 24 hrs and the expression of U90926 was measured via qRT-PCR. **p<0.001 vs cells of non-infected control (Ctrl).

The lab previously performed a genome-wide transcriptome analysis of *C. parvum* infected IEC4.1 cells, transformed by nontumorigenic intestinal epithelial cell line from neonatal mice, and identified a panel of host lncRNA genes whose expression levels are altered during infection (25). Among the lncRNAs that are upregulated is U90926, which is transcribed from the gene locus *U90926* (25). To verify the upregulation of U90926 during *C. parvum* infection, qRT-PCR was performed in IEC4.1 cells following *C. parvum* infection at the 8, 24, and 48 hr time points. There was a significant increase in U90926 expression at 24 hrs post-infection (**Figure 1A**). Using a documented model of intestinal cryptosporidiosis in neonatal mice via oral administration of the parasite (35, 45), we detected infection via immunofluorescent staining, in the villus regions of the ileum from infected animals (**Figure 1B**). U90926 was also upregulated in isolated intestinal epithelium from neonatal mice infected with *C. parvum* at 48 and 72 hrs post-infection (**Figure 1C**). Using an ex vivo infection model employing 2D enteroid monolayers from neonatal mouse ileum (46), we detected a dose-dependent increase of U90926 expression in the infected monolayers (**Figure 1D**).

### Upregulation of U90926 may not be induced through the activation of immune signaling pathways in infected intestinal epithelial cells

**Figure 2:**
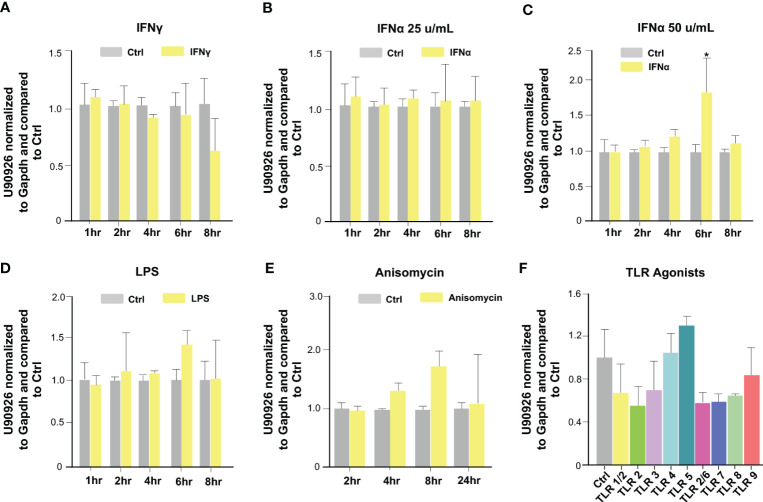
Upregulation of U90926 may not be induced through the activation of immune signaling pathways in infected intestinal epithelial cells. **(A)** Upregulation of U90926 was measured in IEC4.1 cells treated with IFN-γ (10 ng/mL), via qRT-PCR, at time points ranging from 1hr to 8hrs post-treatment. **(B)** IEC4.1 cells were treated with IFN-α (25 u/mL), and samples were collected at time points ranging from 1 to 8 hrs post-treatment. U90926 expression was measured via qRT-PCR. **(C)** IEC4.1 cells were treated with IFN-α (50 u/mL) and collected at time points ranging from 1hr to 8 hrs post-treatment and U90926 was measured via qRT-PCR. **(D)** Expression levels of U90926 were also measured in cells treated with LPS (12.5 ug/mL) and collected 1hr to 8hrs post-treatment. **(E)** IEC4.1 cells were treated with anisomycin (1 ug/mL) and U90926 expression was measured via qRT-PCR. **(F)** IEC4.1 cells were treated with TLR agonists for 16 hr and U90926 expression was measured via qPCR. Data represent means ± SEM from three independent experiments. *p<0.05 vs cells of non-infected control (Ctrl).

We then investigated which host signaling pathways may be responsible for U90926 induction in intestinal epithelial cells following *C. parvum* infection. Previous studies have demonstrated multiple intracellular signals are activated during *C. parvum* infection, such as the NF-κB, JAK/STAT, JNK/MAPK, and type 1 IFN signal pathways (34, 40, 41, 47). IEC4.1 cells were stimulated with IFN-γ (10 ng/mL) and samples were collected at time points ranging from 1h to 8 hr post-treatment. Expression of U90926 was then measured via qRT-PCR. There was no change in U90926 when stimulated with IFN-γ (**Figure 2A**). IEC4.1 cells were also treated with IFN-α (50 u/mL and 25 u/mL) and there was a slight increase in U90926 expression at the 6 hr time point when cells were treated with IFN-α (50 u/mL), however, this modest change was not detected at any other time point or when cells were treated with a lower dose of IFN-α (25 u/mL) (**Figures 2B, C**). In addition, there was no change in U90926 expression in IEC4.1 cells stimulated with LPS or a JNK/MAPK activator anisomycin (48) (**Figures 2D, E**). Treatment with various Toll-like receptor (TLR) agonists, including TLR2, 4, 5, 7, and 9 all of which have been shown to be expressed on IEC4.1 cells (44), did not alter U90926 expression levels (**Figure 2F**). Of note, IEC4.1 cells displayed cellular response to these above stimuli when treated with the indicated doses and time course as reflected by the upregulation of selected genes following each stimulus [e.g., interferon-gamma induced GTPase (*Igtp*) for IFN-γ stimulation] (data not shown). The above data suggest that U90926 expression is increased during *C. parvum* infection but is not controlled by the tested host immune signaling pathways that are activated in infected IEC4.1 cells.

### Impact of U90926 induction on parasite burden in intestinal epithelial cells

**Figure 3:**
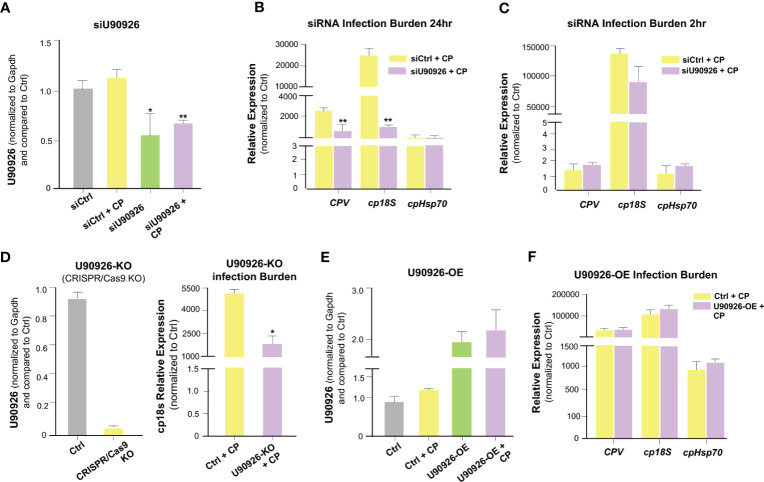
Impact of U90926 induction on parasite burden in intestinal epithelial cells. **(A)** Knockdown of U90926 with a designed siRNA in IEC4.1 cells. Cells were treated with siRNA to U90926 followed by infection with *C. parvum* (CP). U90926 expression was then measured *via* qRT-PCR and a nonspecific siRNA sequence was used as a control. **(B, C)** Impact of knockdown of U90926 on *C. parvum* infection burden. Cells were treated with knockdown U90926 siRNA and exposed to *C. parvum* infection for 24 hr **(B)** or 2 hr **(C)** followed by qRT-PCR to measure parasite infection burden. **(D)** U90926 CRISPR/Cas9 knockout cells (U90926-KO) were infected with *C. parvum* for 24 hrs and infection burden was measured via qRT-PCR. **(E)** IEC4.1 cells were transfected with a plasmid expressing U90926 RNA and infected with *C. parvum*. U90926 was measured via qRT-PCR. **(F)** Cells were transfected with a plasmid expressing U90926 RNA (U9926-OE) followed by infection with *C. parvum*. qRT-PCR was then used to measure infection burden. Data represent means ± SEM from three independent experiments. * p<0.05, **p<0.001 vs cells of non-infected control (Ctrl).

We sought to further explore the relationship between U90926 expression and *C. parvum* infection. We used RNA interference to knock down U90926 in host cells and measure its impact on *C. parvum* infection burden. *C. parvum* 18s (*cp18s*), *Cryptosporidium parvum* virus (*CPV*), and Hsp70 (*cpHsp70*) levels, reflecting parasite burden, were measured at 24 hrs after exposure to *C. parvum* (a time point reflecting activation of the cellular defenses protecting against infection), as well as at 2 hrs after exposure to the parasite (a time point reflecting the initial attachment and invasion of the parasite into host cells) (49). The designed siRNA to U90926 significantly decreased U90926 expression in both non-infected and *C. parvum*-infected IEC4.1 cells (**Figure 3A**). Interestingly, knock down of U90926 resulted in a decrease of *C. parvum* infection burden compared to cells treated with the nonspecific control siRNA after 24 hrs (**Figure 3B**) but no significant change in infection burden after 2h (**Figure 3C**). Additionally, U90926 CRISPR/Cas9 knockout cells (U90936-KO) were infected with *C. parvum* for 24 hrs and the infection burden was measured via qRT-PCR. U90926-KO cells displayed lower levels of *C. parvum* infection burden compared to IEC4.1 cells (**Figure 3D**). Complementarily, cells transfected with a plasmid expressing U90926 (U90926-OE) showed increased levels of U90926 in both *C. parvum* infected and non-infected cells (**Figure 3E**) and a slight increase in infection burden of the parasite after 24 hr (**Figure 3F**). These data indicate that U90926 may be playing a pro-parasitic role during *C. parvum* infection in IEC4.1 cells.

### U90926 regulates the host’s gene expression profile in intestinal epithelial cells following *C. parvum* infection

**Figure 4:**
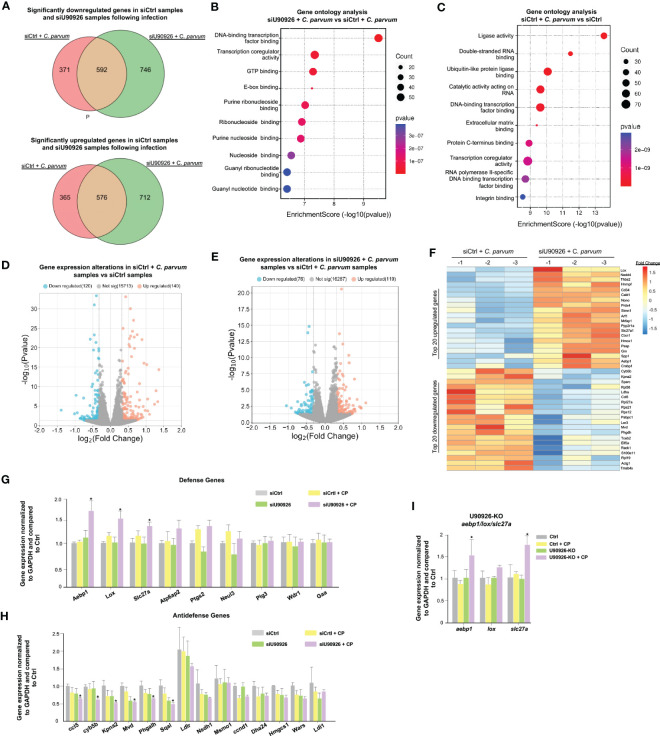
U90926 targets host defense gene expression during *C. parvum* infection. **(A)** Numbers of significantly downregulated and upregulated genes in siU90926 treated samples following *C. parvum* infection compared to siCtrl-treated samples following infection. **(B)** Gene ontology analysis of top 10 molecular functions in siU90926 treated samples following *C. parvum* infection compared to siCtrl-treated samples following infection. **(C)** Gene ontology analysis of top 10 molecular functions in siCtrl treated samples following *C. parvum* infection compared to siCtrl-treated samples. **(D)** Volcano plot of significantly altered genes in siCtrl-treated samples with infection compared to siCtrl-treated samples without infection (adjusted p<0.05 with a fold change >1). **(E)** Volcano plot of significantly altered genes in siU90926 treated samples with infection compared to siCtrl treated samples with infection (adjusted p<0.05 with a fold change >1). **(F)** Heatmap representing top 20 upregulated and top 20 downregulated genes in IEC cells treated with siRNA targeting U90926 followed by *C. parvum* infection. siRNA targeting U90926 was transfected into IEC cells and infected with *C. parvum* followed by genome-wide array analysis. The top 20 upregulated and top 20 downregulated genes are shown, presented as fold changes to the mean value of the log^2^ ratios in the noninfected control (n = 3). **(G)** Upregulation of host defense genes *Aebp1*, *Lox*, and *Slc27a* in IEC cells treated with siRNA targeting U90926 followed by *C. parvum* infection. siRNA targeting U90926 was transfected into IEC cells followed by *C. parvum* infection and expression levels of select defense genes were measured via qPCR and compared to a non-infected scrambled siRNA control. **(H)** Downregulation of host anti-defense genes in IEC cells treated with siRNA targeting U90926. A panel of anti-defense gene expression was measured via qRT-PCR and compared to non-infected samples transfected with a scrambled siRNA. **(I)** Upregulation of host defense genes *Aebp1*, *Lox*, and *Slc27a* in CRISPR/Cas9 U90926 knockout cells during *C. parvum* infection. Data represent means ± SEM from three independent experiments. *p<0.05 vs cells of scrambled siRNA control (Ctrl).

Given its pro-parasitic role in intestinal epithelial cells, we sought to measure the effects of U90926 on the host’s gene expression profile in infected IEC4.1 cells. We used the siRNA interface to knock down U90926 during *C. parvum* infection and performed RNA-Seq analysis to compare the gene expression profiles in samples treated with an siRNA targeting U90926 (siU90926) and those treated with an siRNA control (siCtrl). A total of 1,338 genes were downregulated in the siU90926 samples following infection with *C. parvum* comparable to the 963 genes downregulated in the siCtrl-treated cells following infection (p<0.05). A total of 1,288 genes were significantly upregulated in the siU90926 treated samples following infection and 941 genes were significantly upregulated in the siCtrl samples infected (p<0.05). Among the 1,288 genes significantly upregulated, 576 were common in both the siU90926 samples and siCtrl samples, however, a subset of 712 genes were only upregulated in the siNR_033843 samples (**Figure 4A**). A full list of the upregulated and downregulated genes is provided in **Table S2**. Gene ontology analysis of these genes revealed a broad range of gene categories, distinct to each sample type. The most enriched molecular function pathways in the siU90926 treated samples infected with *C. parvum* compared to the infected siCtrl include DNA-binding transcription factor binding, transcription coregulator activity, and GTP binding (**Figure 4B**). Whereas in the siCtrl-treated samples infected with *C. parvum*, compared to siCtrl samples without infection, the most enriched pathways include ligase activity, double-stranded RNA binding, and ubiquitin-like protein ligase binding (**Figure 4C**). When we compared the expression levels of genes in samples treated with siCtrl, with or without *C. parvum* infection, we found that 260 genes were significantly altered, either downregulated (120) or upregulated (140) (adjusted p<0.05 with a fold change >1) (**Figure 4D**). Whereas when we compared infected samples treated with siU90926 and siCtrl samples with infection, there was a lower number of gene alterations, either downregulated (76) or upregulated (119) (adjusted p<0.05 with a fold change >1) (**Figure 4E**). The top 20 upregulated and downregulated genes are listed in **Figure 4F**. These top upregulated genes include *Aebp1*, *Lox*, and *Slc27a* and the top downregulated genes include *Ccl5*, *Cyb5b*, *Kpna2*, *Mvd*, and *Phagalh* (**Figure 4F**). Notably, the expression levels of a total of 207 genes were either upregulated (87) or downregulated (120) (adjusted p<0.05 with a fold change >1) between uninfected siU90926 treated cells and uninfected siCtrl treated cells (**Figure S1**), suggesting the impact of U90926 knockdown on the basal expression levels of those genes. All sequence data were described in accordance with MIAME guidelines and deposited in the ArrayExpress database (with the accession numbers: E-MTAB-12972). The expression levels of *Aebp1*, *Lox*, and *Slc27a* were all upregulated in IEC4.1 cells treated with U90926 siRNA followed by *C. parvum* infection for 24hrs compared to IEC4.1 cells treated with a control siRNA as further validated by qRT-PCR (**Figure 4G**). Additionally, a number of anti-defense genes were significantly downregulated in cells treated with siU90926 followed by *C. parvum* infection validated by qRT-PCR (**Figure 4H**).

To further explore the relationship between U90926 and the host defense genes *Aebp1*, *Slc27a*, and *Lox*, U90926 CRISPR/Cas9 knockout cells were infected with *C. parvum*, and the expression levels of *Aebp1*, *Slc27a1*, and *Lox* were measured via qRT-PCR. The U90926-KO cells had an increase in *Aebp1* and *Slc27a* expression, but no significant difference was observed in *Lox* expression (**Figure 4I**). Taken together, this finding supports that U90926 may play a pro-parasitic role during infection with *C. parvum* through the suppression of the host’s defense gene expression.

### U90926 is recruited to the promoter regions of several host genes to regulate their transcription

**Figure 5:**
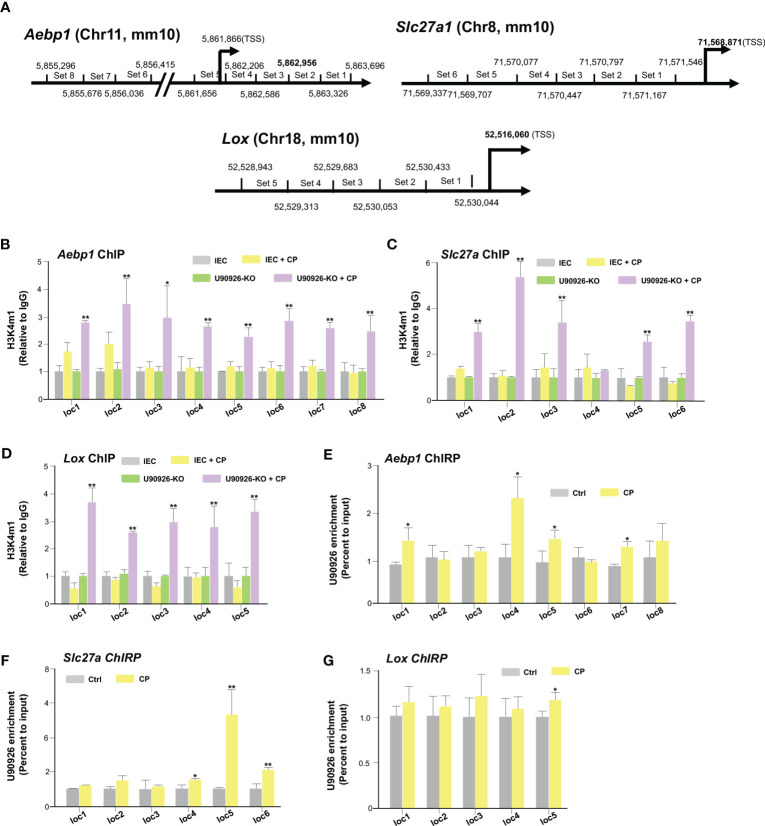
U90926 binds to the promoter regions of several host genes to regulate their transcription. **(A)** Diagram of primer sets of *Aebp1*, *Slc27a*, and *Lox* promoter regions. **(B)** Impact of U90926 on the enrichment of the activation marker H3K4m1 associated with *Aebp1*, **(C)** *Slc27a*, and **(D)** *Lox* gene loci in CRISPR/Cas9 U90926 knockout cells followed by *Cryptosporidium* infection for 24hrs. ChIP analysis was performed using the primer sets as designed. **(E-G)** U90926 recruitment to the *Aebp1* **(E)**, *Slc27a* **(F)**, and **(G)** *Lox* gene loci. IEC4.1 cells were infected with *Cryptosporidium* for 24 hrs followed by ChIRP analysis using a pool of probes specific to U90926 and the PCR primer sets as designed. Data represent means ± SEM from three independent experiments. * p<0.05, **p<0.001 vs cells of non-infected control (Ctrl).

We then sought to examine the impact NR_003483 might have on the transcriptional repression of *Aebp1*, *Slc27a1*, and *Lox* genes during *C. parvum* infection. More specifically, whether U90926 expression impacts the transcriptional active histone modifications associated with *Aebp1*, *Slc27a*, and *Lox* gene loci. By utilizing a ChIP assay, the histone modifications, such as H3K4m1 methylations, that are usually associated with gene transactivation can be examined (50). PCR primers were designed to cover the various regions of regulatory promoters of all three genes (**Figure 5A**). IEC4.1 cells infected with *C. parvum* had increased levels of H3K4m1 in the *Aebp1* gene locus, however, this effect was greatly increased in U90926-KO IEC4.1 cells infected with *C. parvum* (**Figure 5B**). Similarly, U90926-KO cells had increased levels of H3K4m1 in the gene loci of *Slc27a1* (**Figure 5C**) and *Lox* (**Figure 5D**). No change in H3K4m1 levels was observed in IEC4.1 cells at the *Slc27a* gene locus following *C. parvum* infection (**Figure 5C**) and there was a slight decrease in H3K4m1 levels at the *Lox* locus in IEC4.1 cells following *C. parvum* infection (**Figure 5D**).

Furthermore, we examined how the transcription of *Aebp1*, *Slc27a1*, and *Lox* genes is selectively regulated by U90926 and whether this lncRNA is recruited directly to the gene loci. To analyze the occupancy of NR_003483 to the gene loci, ChIRP analysis was performed on IEC4.1 cells following *C. parvum* infection. A pool of biotinylated tiling oligonucleotide probes with an affinity for the U90926 sequence was used to precipitate the chromatin fragments through glutaraldehyde crossing-linking and chromatin isolation. The same PCR primers specific to various sequence regions of the *Aebp1*, *Slc27a*, and *Lox* gene loci for the above ChIP assay were used to identify the DNA sequence of the precipitated chromatin fragments (51). We detected an increased presence of U90926 in the gene loci of *Aebp1* and *Slc27a* in IEC4.1 cells infected with *C. parvum* for 24 hrs (**Figures 5E, F**). There was also an increase in the occupancy of U90926 in the gene locus of *Lox* (**Figure 5G**). Taken together our data indicate that U90926 is recruited to the gene loci of *Aebp1*, *Slc27a*, and *Lox* and may play a role in the inhibition of their transcription.

### Aebp1 is required for epithelial cell-autonomous anti-*Cryptosporidium* defense

**Figure 6:**
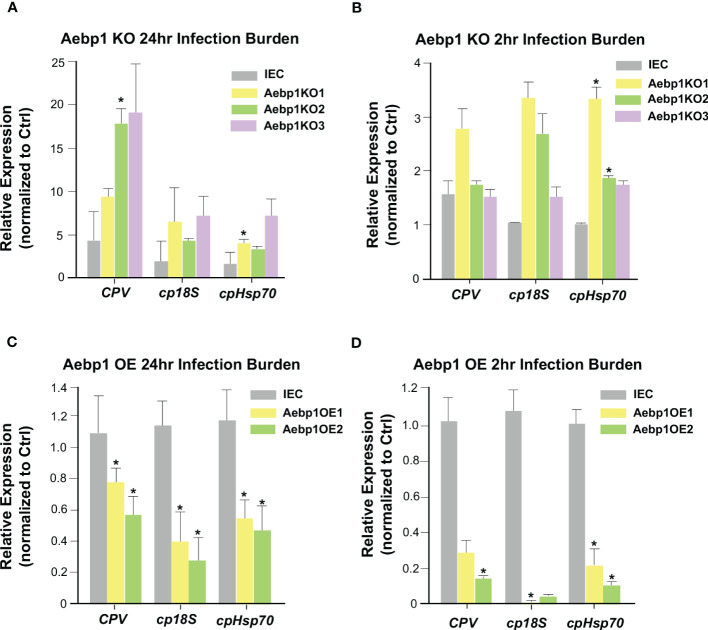
Aebp1 is required for epithelial cell-autonomous anti-*Cryptosporidium* defense. **(A)** Inhibition of Aebp1 results in increased *Cryptosporidium* infection. CRISPR/Cas9 Aebp1 knockout cells were generated (Aebp1KO1-3) and infected with *C. parvum* for 24 hrs and *C. parvum* infection burden was measured via qRT-PCR and compared to the IEC4.1 cell control. **(B)** CRISPR/Cas9 Aebp1 knockout cells showed no significant difference in *C. parvum* attachment/invasion compared to the IEC4.1 control. CRISPR/Cas9 Aebp1 knockout cells were infected with *C. parvum* for 2 hrs and *C. parvum* infection burden was measured via qRT-PCR and compared to the IEC4.1 control. **(C)** Aebp1 overexpression results in a decrease in *C. parvum* 24 hrs infection burden. CRISPR/Cas9 Aebp1 overexpression cells (Aebp1OE1-2) were infected with *C. parvum* for 24 hrs and *C. parvum* infection burden was measured via qPCR and compared to the IEC4.1 control. **(D)** Aebp1 overexpression results in a decrease in *C. parvum* 2 hrs infection burden. CRISPR/Cas9 Aebp1 overexpression cells were infected with *C. parvum* for 2 hrs and *C. parvum* infection burden was measured via qPCR and compared to the IEC4.1 control. Data represent means ± SEM from three independent experiments. * p<0.05 vs IEC control cells (IEC).

Aebp1 is a ubiquitously expressed transcriptional repressor found in many different tissues and cells and is involved in several biological processes including adipogenesis, macrophage cholesterol homeostasis, and inflammation (52–54). Aebp1 has been shown to induce macrophage inflammatory responsiveness leading to enhanced expression of pro-inflammatory mediators (IL-6, TNF-α, MCP-1, and iNOS) via Aebp1’s potential role to promote NF-κB activity (54). To further explore the relationship between Aebp1 and *C. parvum* infection, we generated a CRISPR/Cas9 Aebp1 KO stable IEC4.1 cell line. The cells were verified as knockout cells via qRT-PCR with primers specific for Aebp1 (**Figure S2A**). These cells were infected with *C. parvum* for 2 or 24 hrs and the infection burden was quantified by measuring expression levels of *C. parvum* genes *via* qRT-PCR. Aebp1 KO cells (Aebp1KO 1-3 strains) showed no significant change in *C. parvum* infection burden after 2 hr of infection (a timepoint for *C. parvum* sporozoite attachment to and entry of host cells) however, after 24 hr infection, Aebp1 KO cells displayed significantly higher levels of *C. parvum* infection burden when compared to IEC4.1 cells infected with *C. parvum* (**Figures 6A, B** **)**. Complementary, we generated CRISPR/Cas9 Aebp1 overexpression stable IEC4.1 cell line as were verified as overexpression cells via qRT-PCR with primers specific to Aebp1 (**Figure S2B**). These overexpression cells were infected with *C. parvum* for 2 or 24 hrs and infection burden was measured via qRT-PCR. In line with the knockout cell data, Aebp1 overexpressing cells (Aebp1OE 1 and 2 strains) showed a significant decrease in *C. parvum* infection burden after 24 hrs compared to the wild-type control. Interestingly, after a 2 hr infection, there was also a significant decrease in infection burden in Aebp1 overexpressing cells compared to the wild-type control cells (**Figures 6C, D**). These data highlight the protective role of Aebp1 during *C. parvum* infection.

### The CSpV1 of *C. parvum* induces the upregulation of U90926 during *C. parvum* infection

**Figure 7:**
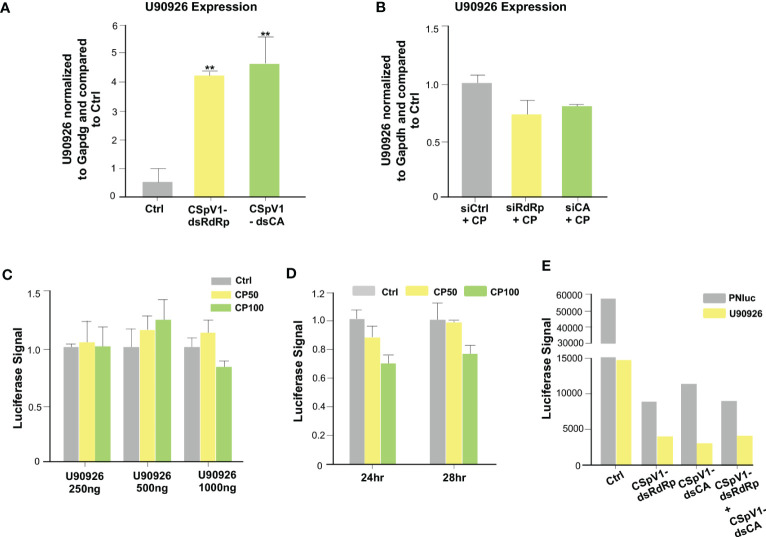
The CSpV1 of *C. parvum* induces the upregulation of U90926 during *C. parvum* infection. **(A)** Transfection of CSpV1-dsRdRp or CSpV1-dsCA increases U90926 expression. CSpV1-RdRp or CSpV1-CP dsRNA was transfected into IEC4.1 cells and U90926 expression was measured via qRT-PCR and compared to a scrambled dsRNA control. **(B)** Transfection of siRNAs to CSpV1-dsRdRp and CSpV1-dsCA suppresses U90926 upregulation during *C. parvum* infection. IEC4.1 cells were transfected with a pool of siRNAs targeting either CSpV1-RdRp or CSpV1-CA followed by infection with *C. parvum* and U90926 expression was measured via qRT-PCR and compared to a scrambled siRNA control. **(C)** Infection with *C. parvum* does not result in an increase in the U90926 luciferase construct signal. A luciferase construct carrying the promoter region of U90926 was transfected into IEC4.1 cells (250 ng, 500 ng, or 1000 ng) followed by *C. parvum* infection. The luciferase signal was measured and compared to a noninfected control. **(D)** No significant change in luciferase signal at 24 hrs or 28 hrs post-infection. The U90926 luciferase construct was transfected into IEC4.1 cells followed by *C. parvum* infection for 24 hrs or 28 hrs. The luciferase signal was measured and compared to a noninfected control. **(E)** Transfection of CSpV1-dsRdRp and/or CSpV1-dsCA does not increase the U90926 luciferase signal. The U90926 luciferase construct, CSpV1-dsRdRp, and/or CSpV1-dsCA were transfected into IEC cells and the luciferase signal was measured and compared to a scrambled siRNA control. Data represent means ± SEM from three independent experiments. **p<0.01 vs control cells (Ctrl).

Proteins and RNAs of parasite origin can be selectively delivered into host cells following *C. parvum* infection (55, 56). In our previous studies, we identified a panel of RNA of parasite origin, as well as the dsRNAs of CSpv1, which are delivered into infected host cells (56, 57). CSpV1 is a dsRNA virus identified in *Cryptosporidium* oocysts from human stool (58, 59). CSpV1 contains two dsRNA segments (CSpV1-dsRaRp and CSpV1-dsCA) that are encapsulated separately and encode for two proteins: the RNA-dependent RNA polymerase (RdRp) and the capsid protein (CA), respectively. We reasoned that the delivery of RNAs of parasite origin is involved in *C. parvum*-induced U90926 expression. We measured U90926 expression levels in IEC4.1 cells transfected with plasmids expressing parasite RNAs that we previously demonstrated to be delivered into infected host cells (56). No obvious induction of U90926 expression was detected in cells transfected with the parasite RNAs tested (**Figure S3**). We then transfected IEC4.1 cells with *in vitro* transcribed CSpV1-dsCA or CSpV1-dsRdRp and examined U90926 expression levels via qRT-PCR. The expression levels of CSpV1-dsCA or CSpV1-dsRdRp were confirmed via qRT-PCR (**Figure S4**). Samples were compared to a scrambled siRNA control. The cells transfected with CSpV1-dsRdRp or CSpv1-dsCA had significantly higher expression levels of NR_003483 than control samples (**Figure 7A**). A pool of siRNAs targeting CSpV1-dsRdRp and CSpV1-dsCA was designed and transfected into IEC4.1 cells followed by infection with *Cryptosporidium*. There was no increase in U90926 in the IEC4.1 cells transfected with siRNA pool targeting CSpV1-dsRdRp and CSpV1-dsCA regardless of *Cryptosporidium* infection (**Figure 7B**), further supporting that CSpV1 is involved in the upregulation of U90926 expression during *Cryptosporidium* infection.

To uncover the mechanism of CSpV1-induced upregulation of host lncRNA U90926, we generated a luciferase construct carrying the putative promoter region (1 kb upstream of start) of U90926. IEC cells were transfected with the U90926 luciferase construct at different concentrations and infected with *Cryptosporidium* (at two separate doses: 50 μl/well or 100 μl/well) and the luciferase signal was measured. Interestingly, there was no significant change in the luciferase signal regardless of the concentration of U90926 luciferase construct transfected into cells or *Cryptosporidium* concentration (**Figure 7C**). Additionally, there was no change in luciferase signal at 24 hr or 28 hr transfection infected with *Cryptosporidium* (**Figure 7D**). We also measured the luciferase signal when IEC4.1 cells were transfected with the U90926 luciferase construct and plasmids expressing CSpV1-dsRdRp or CSpV1-dsCA. We found no increase in the luciferase signal in the samples transfected with U90926 construct and plasmids expressing CSpV1-dsRdRp or CSpV1-dsCA when compared to control cells transfected with an empty vector (**Figure 7E**). Taken together, the data suggest that the upregulation of U90926 during *Cryptosporidium* infection seems to involve CSpV1, however, this may not be due to a transcriptional change in the U90926 promoter.

## Discussion

Cell-autonomous immunity, defined as the ability of a host cell to eliminate an invasive/intracellular infectious agent at the cellular level, is a first line of defense against intracellular microbial pathogens (60). It relies on antimicrobial proteins, specialized degradative compartments, and programmed host cell death (60–63) and is mediated by tiered innate immune signaling networks that sense microbial pathogens and stimulate downstream pathogen elimination programming (61). Upon infection, *Cryptosporidium* sporozoites attach to the apical membrane surface of epithelial cells and form intracellular but extracytoplasmic vacuoles in which the organism remains and develops (6, 11). Intestinal epithelial cell-autonomous immunity is the host’s first defense against *C. parvum* infection.

In a previous study, the lab identified a panel of host cell lncRNAs that are upregulated in intestinal epithelial cells following infection with *C. parvum* (25). Among these host lncRNAs, U90926 was upregulated during infection in IEC4.1 cells, neonatal mice, and 2D intestinal epithelial monolayers. Interestingly, unlike other host lncRNAs identified in the panel whose expression is controlled by host immune signaling, such as the TLR/NF-κB signaling pathway (25, 64, 65), U90926 does not appear to be upregulated in IEC4.1 cells in response to treatment with IFN-γ, IFN-α, LPS, or anisomycin. Furthermore, the inhibition of U90926 via RNA interference in host intestinal epithelial cells resulted in a decreased infection burden of *C. parvum* and the overexpression of U90926 led to an increase in infection burden. This indicates that U90926 upregulation may be beneficial for the parasite during infection of intestinal epithelial cells through suppression of the host’s epithelial cell-autonomous defense mechanisms.

Our data show that U90926 targets several host genes during *C. parvum* infection, including *Aebp1*, *Slc27a*, and *Lox*. Inhibition of U90926 expression during *C. parvum* infection resulted in increased levels of all three genes, suggesting that U90926 is downregulating their expression during infection. lncRNAs function to regulate gene transcription through specific interactions with cellular factors including proteins, DNA, and other RNA molecules (21, 22). In the case of U90926, we found this lncRNA is recruited to the gene loci in the promoter regions of these genes and alters transcriptional active histone modifications.

The *Aebp1* gene encodes for adipocyte enhancer-binding protein-1, a potent modulator of NF-κB signaling and inflammation (66, 67) whereas Lox is a cell surface endocytosis receptor for ox-LDL and recognizes pathogenic microorganisms (68). It is a proinflammatory scavenger protein and is structurally similar to a c-type lectin-like receptor (69, 70). Scl27a is an integral protein involved in the uptake of long-fatty acids into the cell. It has been reported to be important in intracellular signaling and the metabolism of innate immune cells (71–74). Among the three genes, *Aebp1* showed the most drastic difference in expression in the U90926 knockout experiments. It has been shown to induce macrophage inflammatory responsiveness and play a key role in enhancing the expression of pro-inflammatory mediators by promoting NF-κB activity (52–54). In this study, we demonstrated that during *C. parvum* infection Aebp1 knockout cells showed a significantly higher *C. parvum* infection burden compared to control cells. This was complemented by Aebp1 overexpression cells exhibiting decreased *C. parvum* infection burden. Together, our data suggest that U90926 is induced during *C. parvum* infection and suppresses transcription of the *Aebp1* gene. This, in turn, inhibits Aebp1’s cell-autonomous defense role in the intestinal epithelial cells and allows for a robust parasitic infection.

It is interesting that the small dsRNA virus, CSpV1, may be responsible for its upregulation. CSpV1 is a bisegmented dsRNA virus that is found in many *Cryptosporidium* spp. The two genome segments are packaged in separate virions and encode for an RNA-dependent RNA polymerase and a capsid protein (36, 58). CSpV1 seems to be transmitted vertically during the cell division of *C. parvum* cells as well as during gamete fusion of the *C. parvum* replication cycle. No horizontal extracellular transmission has been reported (37). This virus is thought to be associated with avirulent infections of its host and some authors have described *C. parvum* as having a “viral symbiont” (38). There have been few studies looking at CSpV1 and virulence with one study showing a correlation between levels of CSpV1 dsRNA and parasite fecundity (59). However, the cell biology of CSpV1 infection and the host factors that are involved in modulation, inhibition, or are affected by the virus are unknown. We previously demonstrated that several parasite RNAs and CSpV1-dsRNAs are delivered into infected host cells following *Cryptosporidium* infection (56, 57). Our current data indicate that the transfection of CSpV1-dsRdRp and CSpV1-dsCA into IEC4.1 cells leads to an increase in U90926 expression. This effect was inhibited when cells were treated with an siRNA pool targeting CSpV1-dsRdRp and CSpV1-dsCA. We speculate that CSpV1-dsRNAs are upregulating U90926 to target the host genes, including *Aebp1*, and inhibit the immune response to *C. parvum* infection.

In this study, we uncovered a novel strategy by *C. parvum* to evade the host’s immune response by hijacking host lncRNAs to suppress epithelial cell-autonomous antiparasitic defense. Our data also suggest a new role for Aebp1 in intestinal epithelial cell-autonomous defense. Further investigations are required to fully understand how CSpV1-dsRNAs induce U90926 expression and how Aebp1 may carry out its antiparasitic function. It will also be interesting to investigate whether U90926 plays a role during intestinal infection with other microbes.

## Data availability statement

The datasets presented in this study can be found in online repositories. The names of the repository/repositories and accession number(s) can be found below:E-MTAB-12972 (ArrayExpress; https://www.ebi.ac.uk/biostudies/arrayexpress).

## Ethics statement

The animal study was reviewed and approved by The Institutional Animal Care and Use Committees (IACUC) of the Rush University Medical Center.

## Author contributions

MG, ML, and X-MC designed experiments and wrote the manuscript. MG, ML, SD, KJ, and A-YG performed experiments. MG, ML, SW, and X-MC performed data analysis. A-YG and X-MC directed and supervised the study. All authors contributed to the article and approved the submitted version.
