# Supplementary figures and images for: Cryptosporidium parvum hijacks a host’s long noncoding RNA U90926 to evade intestinal epithelial cell-autonomous antiparasitic defense
Source: Front Immunol. 2023 Jun 5;14:1205468. doi: 10.3389/fimmu.2023.1205468 (PMC10280636; doi:10.3389/fimmu.2023.1205468)

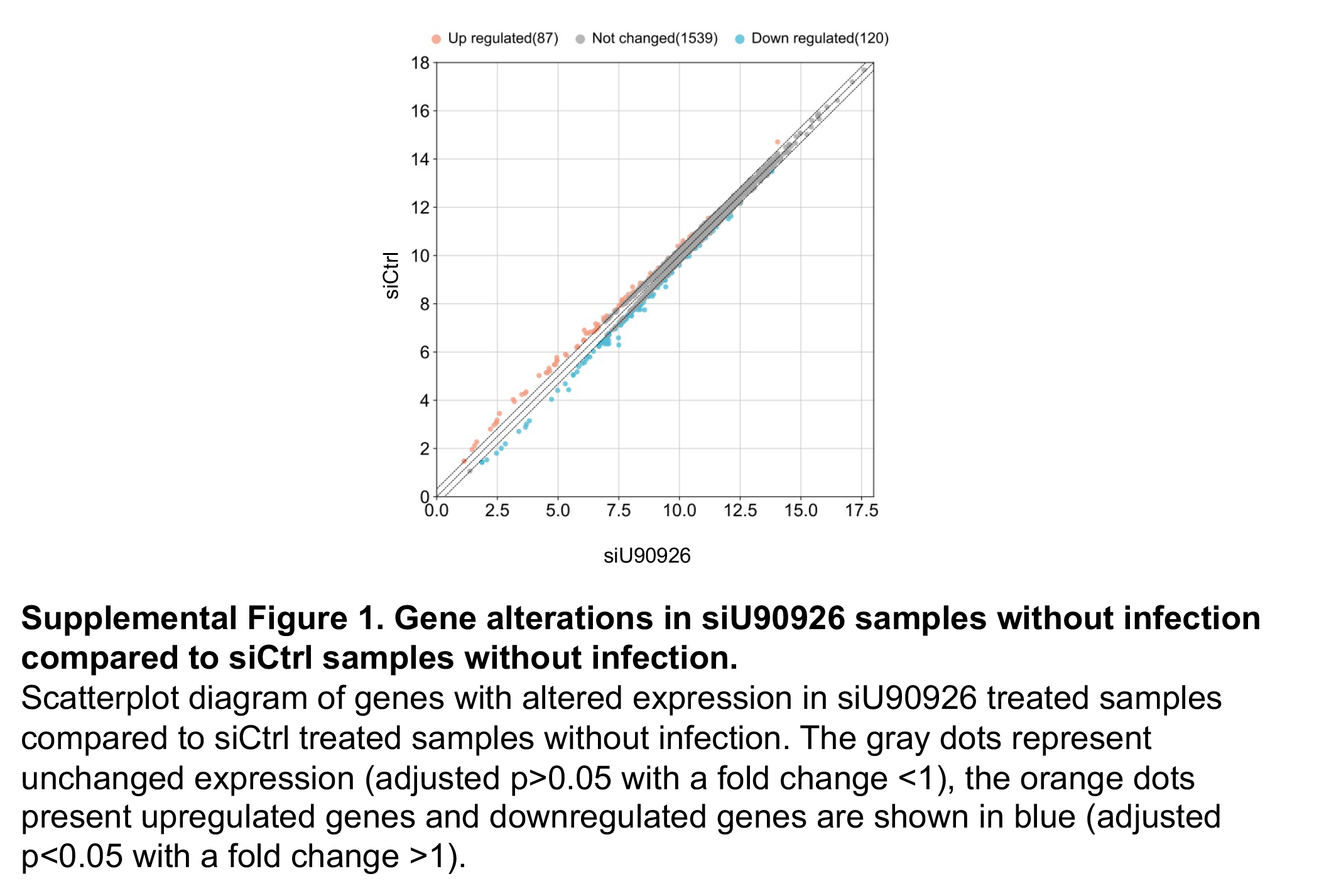

Supplement: Supplementary file 3 [file Image_1.tif]

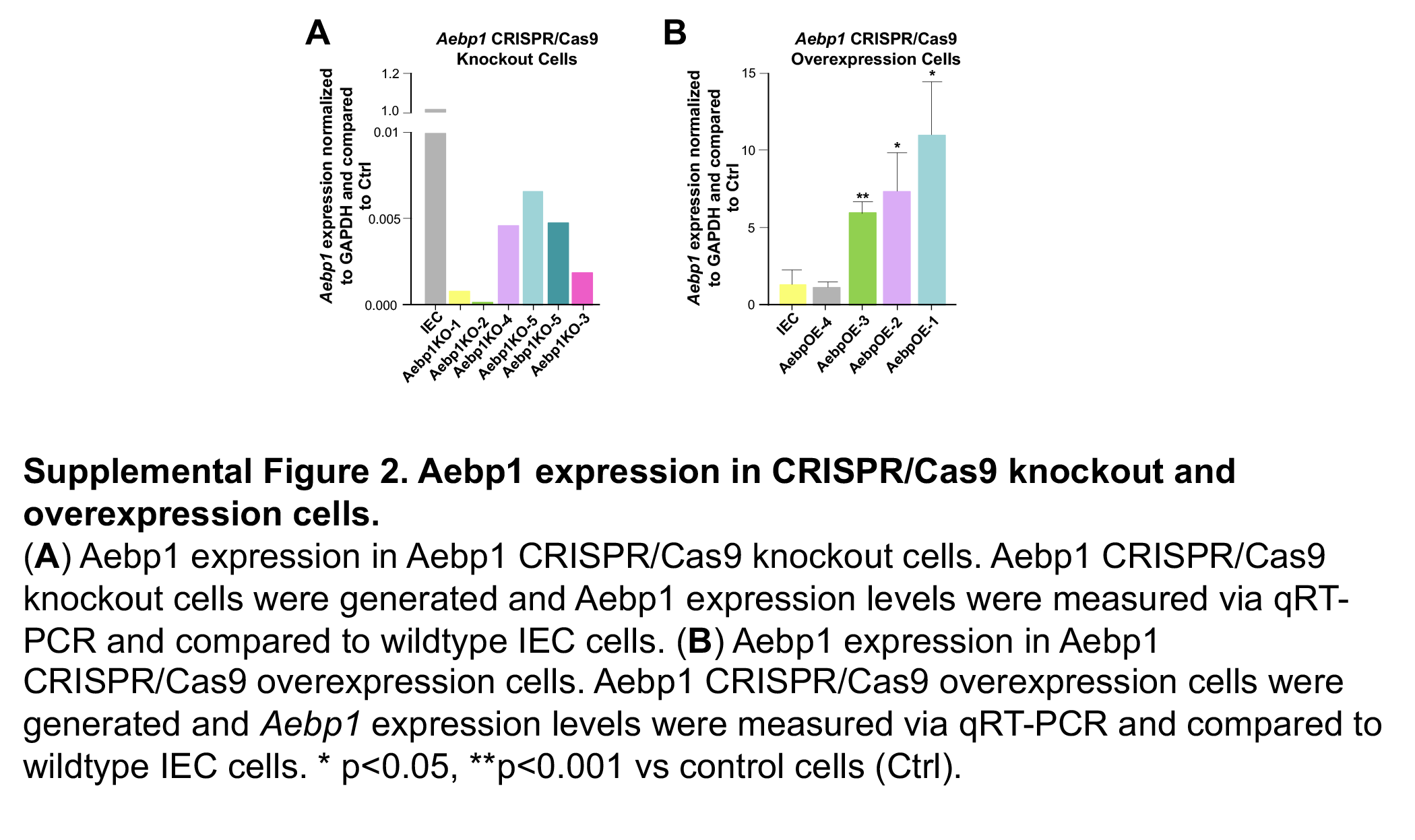

Supplement: Supplementary file 4 [file Image_2.tif]

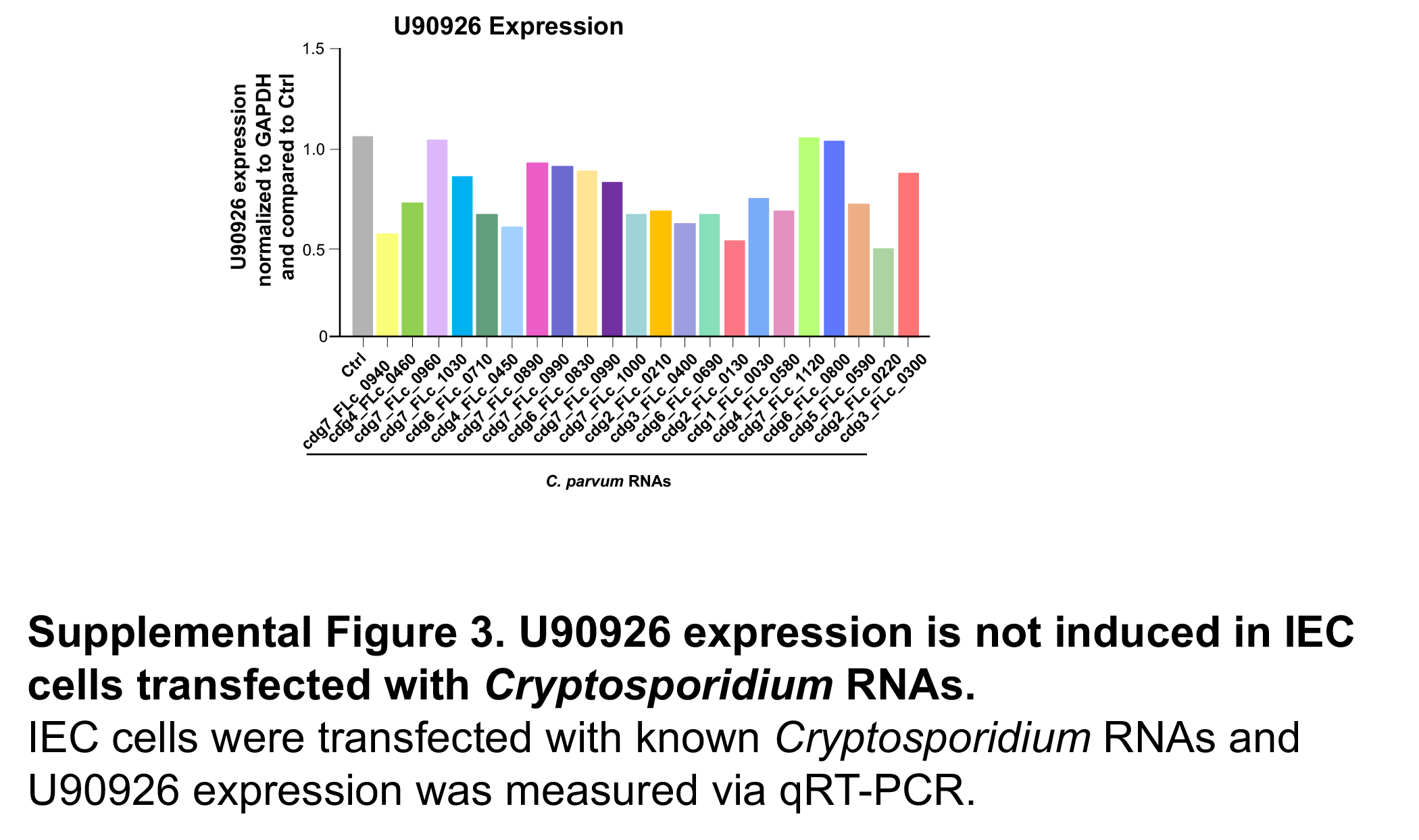

Supplement: Supplementary file 5 [file Image_3.tif]

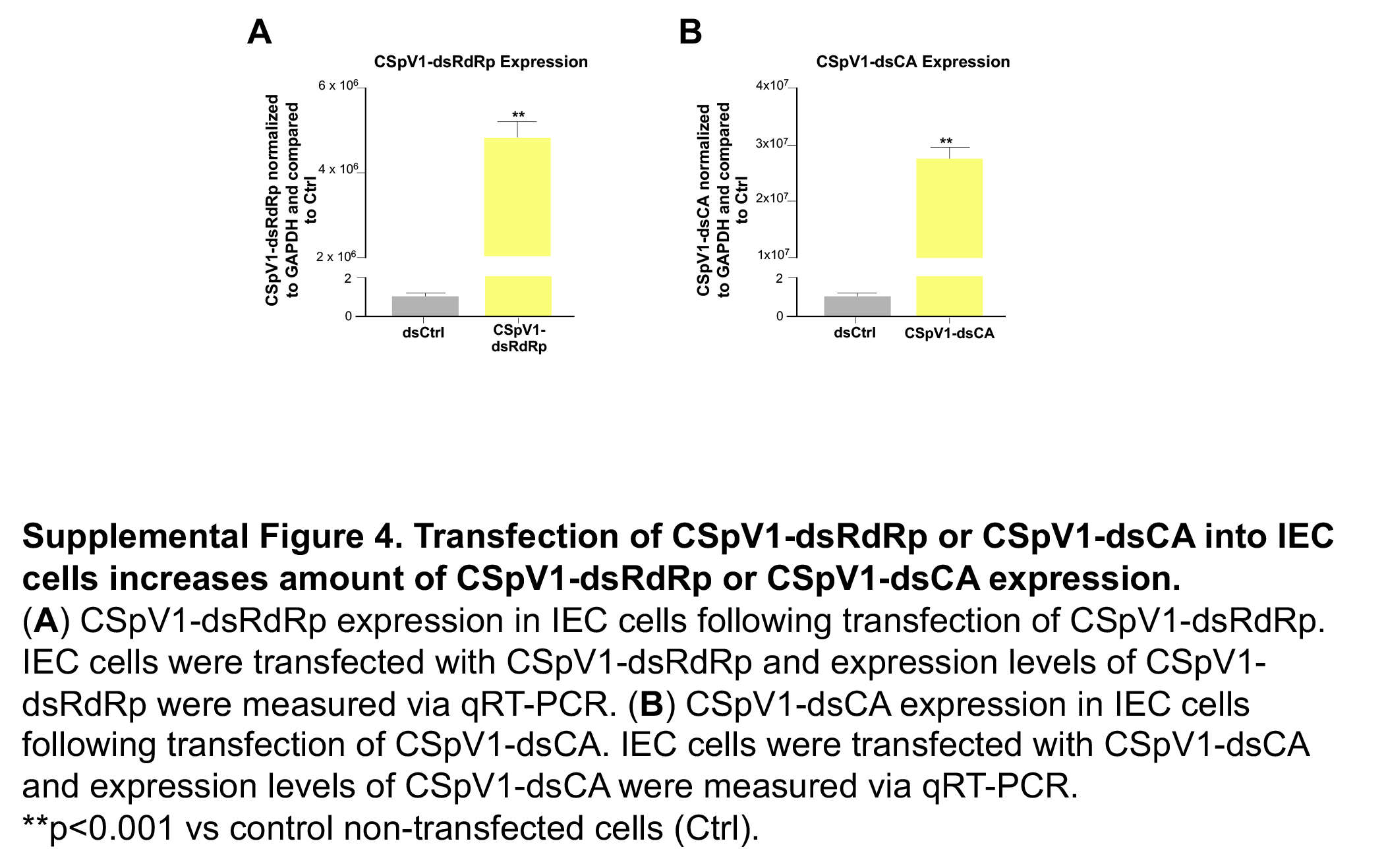

Supplement: Supplementary file 6 [file Image_4.tif]
